# Impact of ablation duration on rhythm outcome after concomitant maze procedure using cryoablation in patients with persistent atrial fibrillation

**DOI:** 10.1186/s13019-017-0620-6

**Published:** 2017-07-24

**Authors:** Dong Seop Jeong, Ji Hoon You, Chang-Seok Jeon, Pyo Won Park, Kiick Sung, Wook Sung Kim, Young Tak Lee

**Affiliations:** Department of Thoracic and Cardiovascular Surgery, Cardiac and Vascular Center, Samsung Medical Center, Sungkyunkwan University School of Medicine, 81 Irwon-ro, Gangnam-gu, Seoul, 06351 South Korea

**Keywords:** Atrial fibrillation, Ablation, Maze operation, Left atrial activity

## Abstract

**Background:**

The aim of this study was to evaluate the impact of ablation duration during a maze procedure using a nitrous oxide-based cryosurgical system.

**Methods:**

From May 2001 to December 2006, 256 consecutive patients who underwent a concomitant maze procedure using nitrous oxide-based cryoablation for chronic atrial fibrillation (AF) during cardiac surgery were enrolled. The ablation duration for each lesion was between 120 s at −60 °C in 140 patients (control group) and 160 s in 116 patients (long duration group).

**Results:**

One in-hospital death occurred, and a permanent pacemaker was implanted in one patient (0.4%). At discharge, absence of AF was noted in 84.5% of the long duration group and in 87.1% of the control group. During follow up, patients in the long duration group achieved and maintained the absence of AF at a higher rate than the control patients (96% vs. 84% at 24 months, respectively: *P* = 0.008). Multivariable analysis identified long AF duration as the only independent predictor of AF recurrence. At late follow up, left atrial mechanical activity was less frequent in the control group than in the long duration group. The mean left atrial volume index was lower in the long duration group than in the control group.

**Conclusion:**

The modified cryomaze procedure is safe and effective. Ablation time plays an important role in achieving and maintaining the absence of AF. Long cryoablation duration is recommended for optimal cryomaze results.

## Background

Atrial fibrillation (AF) is the most common arrhythmia and is also a common condition in patients with heart disease. The maze procedure is a useful modality for sinus rhythm restoration in patients with AF undergoing cardiac valve surgery. The “cut-and-sew” Cox maze procedure was introduced in 1987 and is considered the gold standard for the surgical treatment of AF [1–3]. Although the conventional maze procedure produced excellent results, it has not gained widespread use because of its complexity. For this reason, many surgeons have modified the maze procedure with various energy sources such as radiofrequency, cryoablation, microwave, ultrasound, and laser energy. In particular, the cryomaze procedure has an excellent clinical safety record [4, 5]. However, the most appropriate ablation duration for achieving long-term isolation has not been definitely established. The purpose of this study was to evaluate the effect of ablation duration on cryomaze rhythm outcomes.

## Methods

### Study population

A total of 256 consecutive patients were enrolled between May 2001 and December 2006 for this study. All patients underwent the cryoablation maze procedure concomitant with cardiac surgery for persistent or long-standing AF. Patients with paroxysmal AF were excluded. Additional exclusion criteria for the cryomaze procedure included: a very large left atrium (LA) over 80 mm, atrial calcification, and significant tricuspid regurgitation associated with severe right atrial enlargement. Patients with successful rhythm control were defined as those who recovered normal sinus rhythm within a window period of 3 months after the maze operation and showed no evidence of AF recurrence thereafter, irrespective of anti-arrhythmic agent use during the follow-up period. The Institutional Review Board of Sungkyunkwan University School of Medicine approved this study, and the requirement for patient consent was waived.

### Operative procedures

**Fig. 1:**
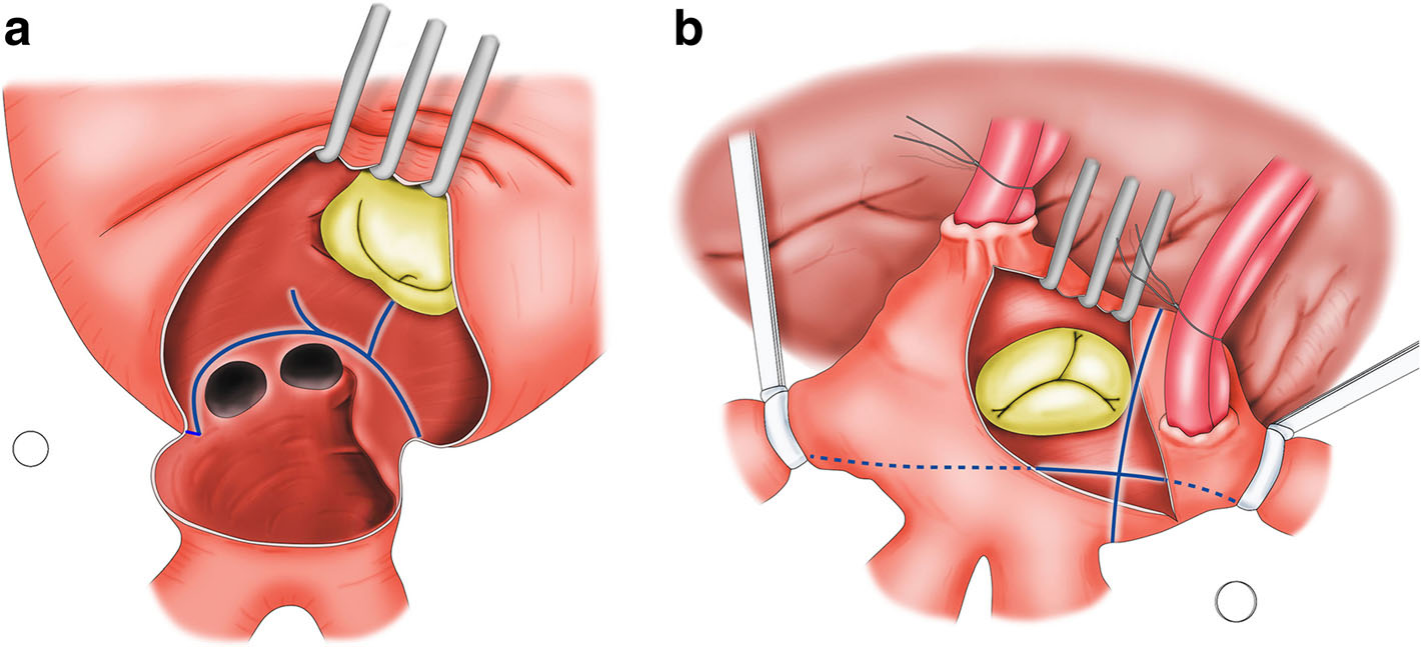
Operative scheme of the modified maze procedure using cryoablation. *Left* atrial procedure (*Blue line*: cryoablation) (**a**) and *Right* atrial procedure (*Blue line*: cryoablation) (**b**)

The modified Cox maze procedure using cryoablation was performed according to published principles and procedures [6]. Briefly, cryoablation lesions were created endocardially with custom-made straight (6 cm) and T-shaped (4 cm) probes (Frigitronics, Cooper Surgical). Cryogenerators were set at −60 °C in both groups and the ablation duration was determined according to the surgeon’s discretion. The LA box and connecting lesions into the posterior mitral annulus were made. The reduction of LA dimensions by partial resection of the atrial wall has been performed since 2005. The left atrial reduction was performed as often as possible, i.e. whenever the LA was greater than 60 mm on preoperative echocardiography. The LA appendage was obliterated internally using a running suture with 4–0 prolene. Other techniques such as external ligation, external stapled exclusion, stapled excision, or combinations of techniques were not performed. A right atrial isthmus lesion and a lesion from the superior vena cava to the inferior vena cava were created (Fig. 1). We occasionally added a lesion from the right atrial appendage to the tricuspid valve annulus in cases with a dilated right atrium. Modified ultrafiltration was used routinely.

### Postoperative management and follow-up

Oral amiodarone (200 mg/day) was administered postoperatively for 2 to 3 months and then withdrawn if there was no AF recurrence. If patients exhibited AF during their hospital stay, 20 to 40 mg/h of amiodarone was given intravenously without loading, followed by oral amiodarone at 200 mg twice a day and then 200 mg/day. Electrical cardioversion was used only in patients with atrial flutter-fibrillation. After 3 to 6 months, oral anticoagulation was withdrawn in the absence of thrombogenic risk factors such as stroke history and mechanical valve replacement. Standard 12-lead electrocardiography was assessed at 1, 3, 6, 9, and 12 months postoperatively and every 6 months thereafter. If patients complained of symptoms suggestive of AF recurrence at any time, electrocardiography and 24-h Holter monitoring were repeated.

Transthoracic echocardiography was performed prior to surgery, before discharge, and during follow-up (3 to 6 months and then 1, 3, 5, and >7 years postsurgery). Left ventricular (LV) end-systolic and diastolic dimensions were obtained from the parasternal view based on the American Society of Echocardiography guidelines [7]. The LV ejection fraction was calculated using Simpson’s method from the parasternal view. The LA dimensions were measured in M-mode tracing from a parasternal long-axis view. The LA volumes were assessed using the ellipsoid (length-diameter) method and corrected to body surface area (LA volume index, LAVI). The transmitral peak velocities of the early and late filling waves were measured in the apical 4-chamber view. The absence of a late filling wave on the tracings was considered to indicate the absence of mechanical atrial contraction.

### Statistical analysis

All statistical analyses were performed using PASW Statistics 19 software (SPSS Inc., Chicago, IL, USA). For comparisons between the two groups, the χ^2^ test or Fisher’s exact test was used for categorical variables, and the unpaired Student’s *t* test was used for continuous variables. Cox proportional hazards regression analysis was performed to identify predictors of outcomes. Covariates that were entered into the model included age, sex, NYHA functional class, diabetes status, hypertension, duration of AF, prior stroke, renal function, serum parameters, and preoperative echocardiographic data. If the *P* value of a covariate was <0.20 in univariate analysis, it was entered into a multivariate analysis model. *P* values <0.05 were considered significant.

## Results

### Patient characteristics

The preoperative patient characteristics are shown in Table 1. Cryoablation was applied for 120 s in 140 patients (control group) and for 160 s in 116 patients (long duration group). No intergroup differences were found, except for the incidence of diabetes. The patient operative data are listed in Table 2. Mitral valve procedures were performed in 91.8% of all patients (235/259). No intergroup differences were observed with respect to cardiopulmonary bypass time or aortic cross clamp time.

**Table 1:** Patient baseline characteristics

	Control	Long duration	*P*-value
	(*n* = 140)	(*n* = 116)	
Age, y	54 ± 12	55 ± 13	0.356
Female sex, n (%)	78 (56%)	74 (64%)	0.190
Mean AF duration, months	47 ± 58	37 ± 43	0.242
AF duration ≥ 10 years, n (%)	16 (11%)	11 (10%)	0.614
NYHA class III-V, n (%)	88 (63%)	68 (59%)	0.335
History of stroke, n (%)	20 (14%)	23 (20%)	0.238
Diabetes, n (%)	9 (6%)	16 (14%)	0.048
Coronary artery disease, n (%)	8 (6%)	12 (10%)	0.169
Hypertension, n (%)	29 (21%)	26 (22%)	0.742
Redo operation, n (%)	8 (6%)	2 (2%)	0.118
Echocardiographic data			
Left atrial diameter, mm	56 ± 8	58 ± 9	0.090
LVEF, %	56 ± 9	59 ± 9	0.051
TR grade, moderate to severe	17 (12%)	13 (11%)	0.971

*NYHA* New York Heart Association functional class, *AF* atrial fibrillation, *LVEF* left ventricular ejection fraction, *TR* tricuspid regurgitation

**Table 2:** Patient operative data

	Control	Long duration	*P*-value
	(*n* = 140)	(*n* = 116)	
Mitral valve procedure			
MVR	78 (56%)	65 (56%)	0.959
MVP	55 (39%)	37 (32%)	0.220
Aortic valve procedure	46 (33%)	34 (29%)	0.542
Tricuspid valve procedure	122 (87%)	94 (81%)	0.180
ASD closure	10 (7%)	6 (5%)	0.517
CABG	1 (1%)	6 (5%)	0.049
Single valve procedure			
MVP	3	7	
MVR	2	3	
AVR	3	7	
TVR / TVP	2	3	
Double valve procedures			
MVP + TVP/TVR	34	25	
MVP + AVP/AVR	3	0	
MVR + TVR/TVP	51	40	
MVR + AVR/AVP	6	2	
AVR/AVP + TVP/TVR	1	0	
Triple valve procedures			
MVP + TVP + AVP	7	4	
MVR + AVR + TVR/TVP	19	20	
AVR + TVP + MVP	8	1	
Left atrial reduction	36 (25%)	42 (36%)	0.077
Miscellaneous	1	4	
CPB time (min)	152 ± 48	143 ± 45	0.129
ACC time (min)	131 ± 91	115 ± 36	0.076

*MVR* mitral valve replacement, *MVP* mitral valve plasty, *ASD* atrial septal defect, *CABG* coronary artery bypass grafting, *AVR* aortic valve replacement, *AVP* aortic valve plasty, *TVR* tricuspid valve replacement, *TVP* tricuspid valve plasty, *CPB* cardiopulmonary bypass, *ACC* aortic cross-clamp, *LA* left atrium

### Postoperative outcomes

One in-hospital death occurred (0.4%). The patient, who required preoperative extracorporeal membrane oxygenator support, died after left ventricular volume reduction surgery for dilated cardiomyopathy that developed after atrial septal defect closure. Five patients underwent cardioversions to restore sinus rhythm against typical atrial flutter (*n* = 3) and early recurrence of atrial fibrillation (*n* = 2). Permanent pacemaker implantation was required in one patient due to sick sinus syndrome. The early postoperative data are summarized in Table 3. There was no significant difference in intensive care unit days or hospital stay between the two groups. Postoperative complications included low cardiac output syndrome requiring extracorporeal membrane oxygenator in 2 patients (0.8%). Re-exploration for bleeding and wound infection occurred in five (1.9%) and six patients (2.3%), respectively.

**Table 3:** Patient postoperative results

	Control	Long duration	*P*-value
	(*n* = 140)	(*n* = 116)	
ICU stay (days)	1.9 ± 2.5	2.0 ± 1.9	0.580
Hospital stay (days)	12 ± 7	12 ± 10	0.743
In-hospital mortality, n (%)	1 (1)	0	0.547
Complications, n (%)			
Postoperative bleeding	5 (4)	0	0.066
Wound infection	2 (2)	4 (3)	0.415
Pacemaker implantation	0	1 (1)	0.453
Acute kidney injury	3 (2)	1 (1)	
IABP or ECMO	1 (1)	1 (1)	0.999
CVA	0	0	0.999
Cardioversion	1 (1)	4 (3)	0.179

ICU intensive care unit, *IABP* intra-aortic balloon pump, *ECMO* extracorporeal cardiopulmonary support, *CVA* cerebrovascular accident

The overall rate of cardiac-related mortality was 0.2% (4/255); no intergroup difference was observed [2% (3/139) in the control group versus 1% (1/116) in the long duration group; *P* = 0.629].

### Rhythm outcomes

**Fig. 2:**
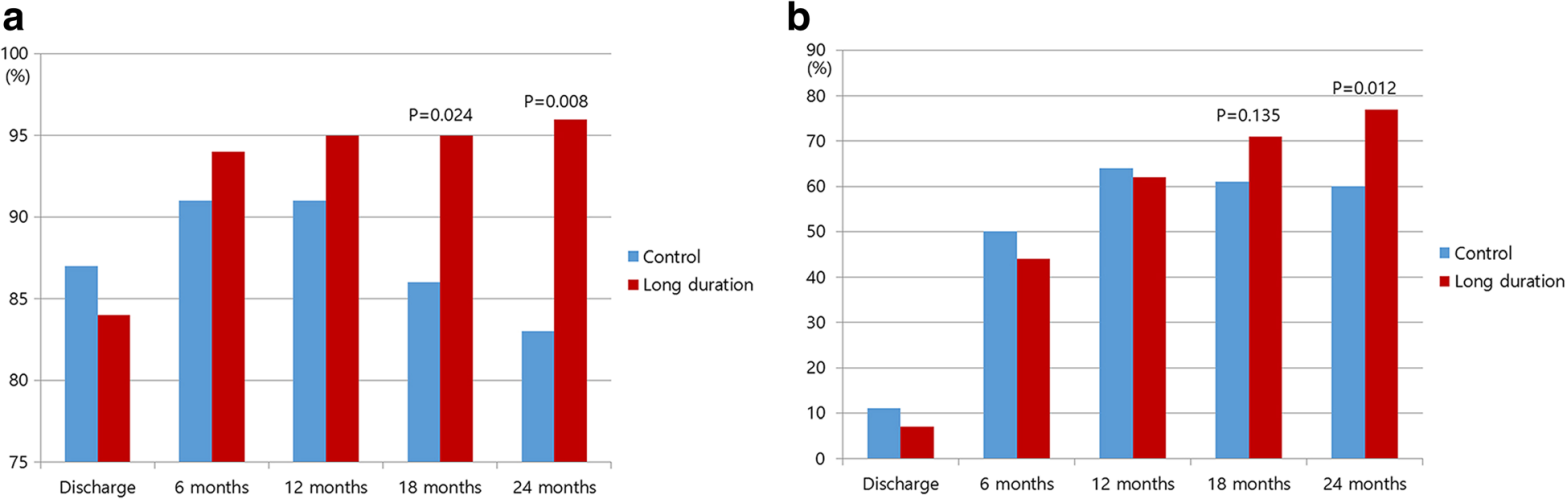
Freedom from atrial fibrillation. All population (**a**) and Off drug population (**b**)

At the time of hospital discharge, sinus rhythm was observed in 87.1% of the control group and 84.5% of the long duration group. Over time, the incidence of sinus rhythm restoration increased in both groups (Fig. 2). However, the rate of freedom from AF recurrence was higher in the long duration group than in the control group at 18 months and at 24 months postsurgery (Fig. 2a). The rate of freedom from off-drug AF recurrence was also higher in the long duration group than in the control group at 24 months postoperation (Fig. 2b).

In the control group, univariate analysis revealed that long AF duration, hypertension, and enlarged left atrium were associated with late AF recurrence. Multivariable analysis identified long AF duration and enlarged left atrium as independent predictors of AF recurrence. In the long duration group, univariate analysis revealed that old age, NYHA functional class, long AF duration, diabetes, and enlarged left atrium were associated with late AF recurrence. Multivariable analysis identified long AF duration as the only independent predictor of AF recurrence (Table 4).

**Table 4:** Predictors of AF recurrence at late follow up according to ablation duration

Variables	Univariate	Multivariate		
	*P* value	*P* value	OR (95% CI)	
Control group				
Age, y	0.041	0.125		
Female sex	0.463			
AF duration, months	0.008	0.016	1.013 (1.002–1.023)	
NYHA class	0.094	0.130		
Diabetes	0.127	0.394		
Hypertension	0.250			
LAD, mm	0.114	0.246		
Long duration group				
Age, y	0.594			
Female sex	0.424			
AF duration, months	0.064	0.049	1.007 (1.001–1.013)	
NYHA class	0.397			
Diabetes	0.205	0.084		
Hypertension	0.044	0.008	1.062 (1.016–1.110)	
LAD, mm	0.310			

*AF* atrial fibrillation, *NYHA* New York Heart Association, *LAD* left atrial diameter, *OR* odds ratio, *CI* confidence interval

### Echocardiographic outcomes

**Fig. 3:**
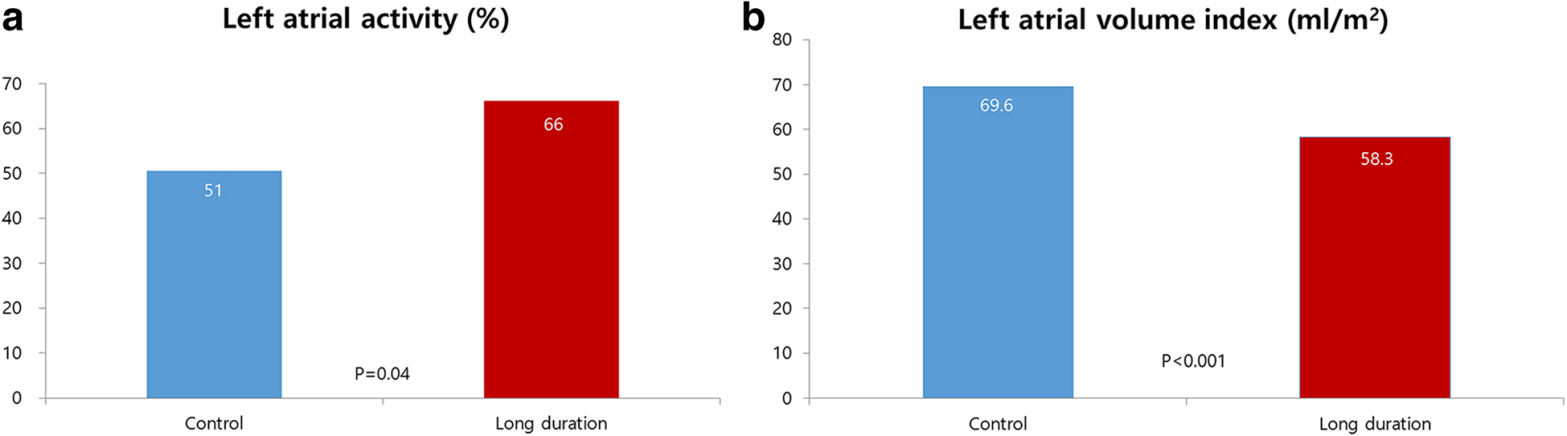
Echocardiographic outcomes in patients with normal sinus rhythm. *Left* atrial activity (**a**) and *Left* atrial volume index (**b**)

Transthoracic echocardiographic follow up findings (median postoperative 91 months) in patients with sinus rhythm maintenance revealed that left atrial mechanical activity was less frequent in the control group than in the long duration group (Fig. 3a). Moreover, the mean left atrial volume index was lower in the long duration group compared with the control group (Fig. 3b).

## Discussion

### Main findings

We investigated the impact of cryoablation time during the maze procedure on clinical and long-term rhythm outcomes. Our main findings are that: 1) Ablation time did not have a significant effect on early postoperative outcomes; 2) the long duration group showed a higher rate of freedom from AF recurrence at 2 years than the control group; 3) the only predictor of AF recurrence was AF duration in both the control group and the long duration groups; and 4) the long duration group had superior outcomes to those of the control group in terms of atrial mechanical activity and left atrial reverse remodeling.

### Impact of cryoablation time on rhythm outcomes

AF is the most common type of chronic arrhythmia in the world, with an estimated prevalence of 0.4%–1% in the general population. AF is associated with an increased long-term risk of stroke, congestive heart failure, and mortality. The maze procedure is a useful modality for sinus rhythm restoration in patients with AF undergoing cardiac valve surgery. The contemporary modified Cox maze III procedure has an excellent success rate for sinus rhythm recovery, up to 90% [8, 9]. Among various energy sources such as radiofrequency, cryoablation, microwave, ultrasound, and laser energy, cryoablation is known to have the benefit of minimal damage to the endocardium and to create homogeneous transmural lesions [5]. The freezing and thawing form intracellular ice crystals, which are lethal to cardiomyocytes and result in irreversible lesions within 2 h after application [10]. By 12 weeks after application, sharply circumscribed, homogeneous, fibrotic full-thickness lesions develop [11, 12]. During nitrous oxide cryoablation, which is the technique used in the present study, the probe is cooled to −60 °C for 1 to 2 min. However, the ablation time needed to reach complete transmural freezing and “ice-ball” formation varies according to several factors such as left atrial wall thickness, degree of left atrial wall fibrosis, and area of the left atrium. Since there is no established protocol for ablation time in cryoablation, the ablation time is at the surgeon’s discretion. Fukada and colleagues [13] applied cryoablation for 1 min at −60 °C. Manasse and colleagues [14] used applications of 2 min (3 min between the inferior left pulmonary vein and the posterior mitral leaflet) at −60 °C. Most reported cryoablation procedures have used applications ranging from 90 s to 2 min at −60 °C or −80 °C [15–17]. In the present study, the long duration group had better outcomes than the control group with respect to sinus rhythm maintenance, both on and off drugs. We recommend that the cryoablation time should exceed 160 s to achieve optimal long-term rhythm outcomes. Further study with longer follow up is mandatory to shed light on the efficacy of long ablation duration for sinus rhythm maintenance.

### Left atrial reverse remodeling and ablation time

The recovery of LA activity after the maze procedure can augment LV filling and stroke volume. Therefore, a lack of LA activity could predispose patients to the development of heart failure by decreasing forward cardiac output and elevating LA pressure. Furthermore, the recovery of LA activity may also contribute to the prevention of stroke or thromboembolism by increasing the blood flow velocity in the LA [18]. Even if sinus rhythm recovers after the maze operation, atrial mechanical function could remain impaired [19]. In a recent study by Buber et al., 47 (31%) of 150 patients who recovered SR after the maze operation showed no evidence of LA mechanical contraction on 3 month follow-up echocardiography [20]. Moreover, the absence of LA contraction resulted in a significant increase in the risk of thromboembolic stroke in patients with sinus rhythm [15]. We hypothesized that the control group would show better left atrial reverse remodeling, including recovery of left atrial activity and reduction of left atrial volume index, once the sinus rhythm was restored because more tissue damage is incurred with longer cryoablation durations. However, interestingly, the rate of left atrial activity recovery was higher in the long duration group than in the control group. Moreover, the left atrial volume index was also lower in the long duration group. One possible explanation is that the control group might have included more silent AF episodes that could be detected in spot eletrocardiography than the long duration group. Further studies using 48 h Holter monitoring or implantable eletrocardiography monitoring are necessary to test this hypothesis.

### Other clinical implications

Nitrous oxide-based cryoprobes are reusable and cost-effective. During the cryomaze procedure, we used two linear cryoprobes, either simultaneously or alternately. One probe was a 15° angled 6 cm custom-made probe. The other was a T-shaped 4 cm linear probe that was custom designed for application at the LA posterior wall and right isthmus. We hypothesize that use of a long cryoprobe might reduce the overall application time. Furthermore, the use of a T-shaped linear probe might facilitate application onto the LA posterior wall, mitral annulus, and right isthmus.

Our modification of the cryomaze procedure is simple and provides excellent results. In particular, we demonstrated a reduced requirement for pacemaker implantation. A pacemaker was implanted in one patient (0.4%) with the diagnosis of sick sinus syndrome, who has since maintained a regular rhythm without a pacing rhythm. The cryolesions resulting from our procedure are separate from the sinus node; this might reduce the risk of sick sinus syndrome due to sinus node devascularization and denervation.

### Study limitations

Our study did have several limitations. First, this study was not a prospective randomized study; therefore, this study was inherently limited by its retrospective nature. However, our cohort is one of the largest to date, with most of the patients treated with the same procedure (cryomaze). In addition, the effect of left atrial activity on sinus/atrioventricular node function or the extent of atrial/perinodal fibrosis was not determined. Therefore, future prospective studies are needed to definitively establish the clinical implications of left atrial activity. The low incidence of permanent pacemaker implantation is another limitation of our study, which might be related to the very strict reimbursement policy in Korea. Our patient group has been followed with a uniform treatment plan (>95%) by a single cardiac surgeon in our institute.

## Conclusion

In conclusion, our modified cryomaze procedure using a long cryoprobe is safe and effective for the treatment of chronic AF. Cryoablation time plays an important role in achieving and maintaining the absence of AF. For this reason, we recommend longer cryoablation durations to achieve optimal cryomaze rhythm outcomes.

## Abbreviation

AF: Atrial fibrillation
LA: Left atrium
LAVI: Left atrial volume index
LV: Left ventricle
NYHA: New York heart association
